# Supplementary material for: Selecting predictive biomarkers from genomic data
Source: PLoS One. 2022 Jun 16;17(6):e0269369. doi: 10.1371/journal.pone.0269369 (PMC9202896; doi:10.1371/journal.pone.0269369)
Supplement: S1 Appendix — Computation of heritability for simulation scenarios. (PDF) [file pone.0269369.s001.pdf]

S1 Appendix

Computation of heritability for simulation scenarios:

We have

- $E(X_j) = 0$, $E(X_j^2) = Var(X_j) = \frac{1}{n-1}$ (due to scaling),
- $E(Z) = 0$, $E(Z^2) = Var(Z) = 1$,
- $Var(X_j Z) = E(X_j^2 Z^2) = \frac{1}{n-1}$,
- $Cov(X_j, X_l) = 0$,
- $Cov(Z, X_j) = 0$, $Cov(Z, X_j Z) = E(Z^2 X_j) = 0$,
- $\beta_j = \beta$ for k/2 indexes and 0 for the rest,
- $\gamma_j = \gamma$ for k/2 indexes and 0 for the rest.

For simplicity, assume that $\beta_j \gamma_j = 0$ for all j. Denote the genetic part of the trait by $G = \sum_{j=1}^{p}(\beta_j X_j + \gamma_j X_j Z)$. The heritability is defined as

$$H^2 = \frac{\text{genotypic variance}}{\text{phenotypic variance}} = \frac{Var(G)}{Var(G) + Var(\mu Z) + \sigma^2}.$$

We can write

$$\begin{aligned} Var(G) &= \sum_{j=1}^{p} \left[\beta_j^2 Var(X_j) + \gamma_j^2 Var(X_j Z)\right] = \frac{k}{2}\beta^2 \frac{1}{n-1} + \frac{k}{2}\gamma^2 \frac{1}{n-1} \\ &= \frac{k}{2(n-1)}(\beta^2 + \gamma^2). \end{aligned}$$

Assuming that $\sigma^2 = 1$ we get

$$H^2 = \frac{\frac{k}{2(n-1)}(\beta^2 + \gamma^2)}{\frac{k}{2(n-1)}(\beta^2 + \gamma^2) + \mu^2 + 1}\ .$$

The proportion of variance for the treatment is apparently

$$H_\mu^2 = \frac{Var(\mu Z)}{Var(G) + Var(\mu Z) + \sigma^2} = \frac{\mu^2}{\frac{k}{2(n-1)}(\beta^2 + \gamma^2) + \mu^2 + 1}.$$
