# Supplementary material for: Selecting predictive biomarkers from genomic data
Source: PLoS One. 2022 Jun 16;17(6):e0269369. doi: 10.1371/journal.pone.0269369 (PMC9202896; doi:10.1371/journal.pone.0269369)
Supplement: S2 Data — The zip archive also includes R Markdown files which were used to obtain summary statistics from the simulation results and a README file which briefly indicates the organization of the simulation files. (ZIP) [file pone.0269369.s005.zip › README R-Code.docx]

**R-Code Simulation_Part1.zip**

Code for the first part of simulations.

- FirstSimulation_rev_Script.R gives the script for the simulations of the final publication
- FirstSimulation_Script.R includes code for the original simulations before the revision

**R-Code Simulation_Part2.zip**

Code for the second part of simulations.

- SecondSimulation_ScriptRevision.R gives the script for the simulations of the final publication
- SecondSimulation_Script.R includes code for the original simulations before the revision

**R-Code Simulation_Part3.zip**

Code for the third part of simulations.

- SNPSimulation_rev_Script.R gives the script for the simulations of the final publication
- SNPSimulation_get_p_eff.R is the code to determine the effective number of SNPs to deal with the correlation patterns

**R Markdown Files for Summaries.zip**

Various R Markdown files to get summaries of results from the different simulations.
