# Supplementary material for: Selecting predictive biomarkers from genomic data
Source: PLoS One. 2022 Jun 16;17(6):e0269369. doi: 10.1371/journal.pone.0269369 (PMC9202896; doi:10.1371/journal.pone.0269369)
Supplement: Attachment — Submitted filename: Rebuttal Letter.pdf [file pone.0269369.s006.pdf]

Rebuttal Letter for "Selecting predictive biomarkers from genomic data"

We want to thank the reviewers and the academic editor for their remarks which have helped us to improve the manuscript. Concerning the remarks of the academic editor we would like to provide the following answers:

1) Simulation of more realistic SNPs that are correlated,

4) Application of the proposed methods to real data,

3) Bonferroni correction for correlated tests,

Answer: We have addressed these three points at once by having provided some additional simulation results based on SNP data which is artificial but has the correlation structure of real SNP data. To this end we simulated an F1 generation starting with real SNP data from two different populations (see Sculz et al. 2017 for further details). The principle motivation underlying this approach is that it is usually very problematic to publish real SNP data due to privacy issues, whereas for our purposes it is sufficient to consider synthetic SNP data with reasonable linkage disequilibrium (LD) patterns. We have used genotype data of 7297 SNPs from one chromosome for a sample of 1000 individuals. This can be made publicly available as a web supplement. Consequently the biomarkers we consider have essentially the same correlation structure as real SNP data. However, we still simulate the trait values to make statistical comparison of the different methods actually possible. The SNP data and the SNPs chosen for the data generating model are extensively described in Appendix 3 (which will become a web supplement). The SNPs show the typical block structure of genomic data from one chromosome.

Concerning the question of Bonferroni correction for correlated tests, one approach is to compute an 'efficient number' of markers which corresponds to the number of independent tests which would provide the same maximal test statistic that one obtains for the correlated test statistics assuming the total null hypothesis. One can then perform a pseudo - Bonferroni correction using that efficient number instead of the total number of tests. This approach was described in full detail in Bogdan et al. (2008). For our synthetic SNP data the estimated efficient number for both prognostic and effective markers is 13700, compared to the total number of 2*7297 = 14594 markers. The pseudo - Bonferroni correction then uses the corrected significance level α / 13700 instead of α / 14594. The effective number can also be used to adapt the penalty of the mBIC2 criterion.

A further difficulty in the case of correlated markers is how to define true positive detections. SNPs which are strongly correlated with causal SNPs give almost the same information in a regression model and thus it is very likely that instead of the correct SNP a highly correlated one might be selected. Particularly in the case of GWAS this is not a big problem, because due to LD strongly correlated SNPs are usually also located in a neighborhood on the chromosome. For this reason it makes sense to consider clusters of SNPs, and classify a detection as true positive whenever it lies within the same cluster as a causal SNP. We used the R package geneSLOPE to cluster SNPs from our data set and we also provide the clustering information together with the genotype data.

2) Identification of prognostic and predictive biomarkers,

Answer: We have added this information in Appendix 2 as supplementary material and concerning the question of reviewer 2 we have specifically added markers in the SNP based simulation which are both predictive and prognostic.

5) Possible overlap with authors' previous publication (Frommlet and Bogdan, 2020).

Answer: There is not really any overlap. More details are provided below as an answer to Reviewer #3.

Additionally we want to mention two important changes we have made which do not relate directly to requests from reviewers:

- We have used a more recent implementation of SLOBE which aims at controlling the FDR at a level of 5%. This gave slightly different results for the first part of our simulation. The new version of SLOBE now does not suffer from a decrease in power for more complex models in the high dimensional case, but instead its type I error increases in that situation. We have changed the text of the manuscript accordingly.

- When rerunning the simulations for treatment efficacy we noticed that we had made some choice which was not exactly wrong but which was also not particularly reasonable. In Method 3, which is designed to test for efficacy in subgroups, we had selected those patients as responders for whom the predictive index was positive. This is exactly the method we had described in the manuscript. However, after sample splitting the power to detect predictive biomarkers in the training sample is relatively low and particularly for k = 2 it is very often the case that no predictive marker is selected at all. In this cases the estimated predictive index only depends on the estimated treatment effect μ and thus becomes constant for all individuals. If that estimate is larger than 0 it thus means that all the individuals are selected as responders, which is really not what we would like to achieve with this method. We have now rectified this flaw and set the p-value for the test on efficacy in the subgroup always to 1 when no predictive marker was selected in the training sample. Accordingly the power for Method 3 to detect efficacy in a subgroup has substantially decreased for small models. On the other hand for the more complex models with larger heritability the results have remained more or less identical and thus qualitatively the main message of our manuscript has not changed.

Reviewer #1

There were no specific comments by reviewer #1 to be answered.

Reviewer #2

1. It is assumed all the SNPs are independent, while in reality the multicollinearity of neighboring SNPs makes them highly correlated. The Bonferroni correction is overly conservative in association studies in which the tests are correlated. Adaptive LASSO and SLOBE have advantages with correlated covariates. I am wondering what's the performance of the proposed methods with correlated SNPs.

Answer: For prognostic markers we studied the question of correlated SNPs already in the past (see Frommlet et al., 2011, and Dolejsi et al. 2014) with respect to single marker tests and mBIC2 based selection. However, we have not directly compared these methods with adaptive LASSO and SLOBE in SNP data. Hence we have followed the suggestion of the reviewer and have performed additional simulations for biomarker identification where we are using real SNP data for our design matrix. The simulation procedure is then very much the same as in the first part of the simulation study in our original manuscript.

To keep the computational effort reasonable we have used only the genotype data of 7297 SNPs from one chromosome of 1000 individuals. The data stem from an admixture population and were generated as described in full detail by Sculz et al. (2017). Based on real SNP data from two different populations an artificial admixture population was generated as an F1 generation. This results in genotype data which have very much the same correlation structure as real SNP data but which do not stem from real persons and therefore can also be made publicly available (which is the policy of PLOS ONE) without running into difficulties of privacy.

2. In the simulation, half of the causal variants were prognostic and the other half predictive. So there is no overlap in the biomarkers for main effect and interaction with dose. In reality a biomarker can be both prognostic and predictive. I am wondering if the proposed methods can identify both effect and what's the performance.

Answer: As we are using a linear model there really should not be any issue with respect to identifying markers which are both prognostic and predictive at the same time – specifically as long as we are using standardized markers and a balanced design for the treatments. In that case the main effects (for the prognostic markers) and the interaction effects (for the predictive markers) are more or less orthogonal and there is no specific reason why there should be any problem in identifying markers which are both prognostic and predictive. To demonstrate this points we included 5 markers in the new simulation scenario with SNP data which are both prognostic and predictive. Apparently things become more difficult for an unbalanced design where the two treatment groups are not allocated an equal number of individuals, but this is under the control of the researcher who designs a trial and problems of this kind can thus be avoided.

3. For biomarker identification, Fig 1 doesn't distinguish between prognostic and predictive biomarkers. However, predictive biomarkers play a role in the personalized treatment selection and are of interest in clinical settings. It would be good to know the selection of predictive biomarkers.

Answer: We have now included the information about the identification rates for prognostic and predictive markers as supplementary material. We were using in our simulations the same effect sizes for prognostic and predictive markers and hence it is not surprising that we got also more or less the same behavior in terms of statistical power and type 1 error rates. In case of simulations with SNP data the situation is a bit more complex and we discuss in detail the power to detect individual SNPs (purely prognostic, purely predictive or both). Here the main difference seems to come depend on specific correlations of the causal SNP with other SNPs and not so much on the fact whether a SNP is prognostic, predictive, or both.

4. From part 1 results, mBIC2 has best performance under different scenarios and is adopted in part 2. Method 1, 2, 2a tested treatment effect in whole population, method 3 & 4 tested treatment effect in predicted responders R(X)>0, and method 5 & 6 tested treatment effect overall or in subgroup. They are different hypotheses and methods within each hypothesis are comparable. It is confusing to compare method 2 vs method 4 and put all methods in one figure.

Answer: The reviewer is perfectly right, that different hypotheses are tested. However, the purpose of our comparison here is really to assess the success of different experimental designs to identify effective treatments under different scenarios. The researcher does not know in advance whether the treatment works for the whole population or whether there is a subgroup in which the treatment is effective. We would like to offer an approach which allows to make best use of the biomarker data to identify treatments which either work on the whole population or which are at least beneficial in

some subgroup which can be identified with predictive biomarkers. For that reason we would really like to keep Figure 4 the way it is and compare the different strategies.

However, we agree that it might be unnecessarily confusing to have two different methods for testing within the subpopulation of responders – particularly as we did not observe a large gain in power there. We have thus removed methods 4 and 5 from Figure 4. We hope that this helps to make the Figure more transparent. Furthermore we have made it more explicit in the text that different hypotheses are tested by the different methods and that power refers to the successful identification of treatments which are effective in a general sense – which means either for the whole population or within some subgroup.

5. When test treatment effect overall or in subgroup, each test is performed at $\alpha=0.025$ with Bonferroni correction. Those two tests are correlated and Bonferroni correction might be too conservative. For the adaptive clinical trial as in the discussion, different α-spending functions can be used and should be discussed.

Answer: This is an interesting point for further research but we have the feeling that investigating this point in this manuscript would distract from our main message. We have therefore addressed this point only briefly in the discussion. Apparently what might help is to make use of the inherent correlation structure between the test based on the whole population and the test in the subpopulation due to overlapping data points. We have provided references which point in the direction how to address this issue. We have also provided further references concerning the question on alpha spending for a potential adaptive design.

6. SLOBE and mBIC2 are compared with adaptive LASSO, it would be helpful to provide the equation of adaptive LASSO too and compare with equation (3) SLOPE.

Answer: We have followed this suggestion and included a mathematical description of the specific version of the adaptive LASSO that we used for our simulations as well as the reference [Kraemer et al. (2009)] where the specific method is described in full detail.

Minor comments:
The resolution of the figures needs to be improved.

Answer: We have increased the resolution of the figures. We hope that this is now sufficient. Perhaps the problem might also be connected with the way PLOS ONE provides the figures in the system. You might have to download the Figures before getting the actual version. The figure provided on the overview page about the submitted article tend to be rather blurry.

Reviewer #3

1. This paper needs to be rewritten as many terms are not introduced at all. For example, I am not sure what are ‘prognostic index’, ‘Treatment Efficacy’, etc.

Answer: We apologize that we had four instances where we erroneously used the expression ‘prognostic index’. These were typos. In all instances it should have been the ‘predictive index’ like everywhere else in the manuscript. ‘Treatment efficacy’ is a rather standard term in the medical literature to denote that a treatment is effective under standardized conditions. We have given some

additional comment in the text to point this out to readers who are not so familiar with the medical literature and we have also made it more transparent that we are interested to investigate two different questions, efficacy in the overall population and efficacy in a subgroup specified by the biomarkers. We have checked the manuscript carefully and did our best to further improve readability where necessary.

2. In addition, no real-world genomic data were used in this paper but only some simulated SNPs. This paper will be greatly improved if the authors can apply the machine learning models directly to human genomics data. There are many published human genomic datasets in the GEO database, the authors can take advantage of them.

Answer: A similar request was made by reviewer #2 and thus we have followed this suggestion. However, as far as we can see the GEO database only includes gene expression data but no SNP data. We have therefore used SNP data as described in detail in Sculz et al. (2017). The resulting artificial genotype data looks very much like real SNP data but there are no privacy issues and the data can be made publicly available (which is the policy of PLOS ONE). In Appendix 3 which will serve as a web-supplement we have described the genotype data set in full detail.

3. The content of this paper seems to overlap with the authors' other paper (Frommlet and Bogdan, 2020) titled 'Identifying important predictors in large data bases - multiple testing and model selection'.

Answer: There is really hardly any overlap between this manuscript and the other document that the reviewer refers to. 'Identifying important predictors in large data bases - multiple testing and model selection' is a book chapter of the forthcoming Handbook of Multiple Testing. It gives a very general overview of different model selection procedures which can be used in high dimensions and as such also discusses information criteria like mBIC2 and L1 based selection procedures like SLOBE. However, the chapter of the handbook is not at all concerned with the question of predictive biomarkers and with the question of how to use biomarkers to identify subgroups of patients for whom a treatment is beneficial, which are the main topics of this manuscript.

References:

Bogdan, M., Frommlet, F., Biecek, P., Cheng, R., Ghosh, J.K. and Doerge, R.W., 2008. Extending the Modified Bayesian Information Criterion (mBIC) to dense markers and multiple interval mapping. Biometrics, 64(4), pp.1162-1169.

Frommlet, F. Ruhaltinger, P. Twarog, M. Bogdan, *Modified versions of Bayesian Information Criterion for genome-wide association studies*, 2012, CSDA, 56, 1038 – 1051

E. Dolejsi, B. Bodenstorfer, F. Frommlet, *Analyzing Genome-Wide Association Studies with an FDR Controlling Modification of the Bayesian Information Criterion,* 2014, PLoS ONE 9(7): e103322. doi:10.1371/journal.pone.0103322

N. Kraemer, J. Schaefer, A.-L. Boulesteix (2009) "Regularized Estimation of Large-Scale Gene Regulatory Networks using Gaussian Graphical Models", BMC Bioinformatics, 10:384

P. Szulc, M. Bogdan, F. Frommlet, H. Tang: *Joint Genotype- and Ancestry-based Genome-wide Association Studies in Admixed Populations*, 2017, Genetic Epidemiology 41(6): 555-566. doi: 10.1002/gepi.22056.
