# Supplementary material for: Selecting predictive biomarkers from genomic data
Source: PLoS One. 2022 Jun 16;17(6):e0269369. doi: 10.1371/journal.pone.0269369 (PMC9202896; doi:10.1371/journal.pone.0269369)
Supplement: S2 Data — The zip archive also includes R Markdown files which were used to obtain summary statistics from the simulation results and a README file which briefly indicates the organization of the simulation files. (ZIP) [file pone.0269369.s005.zip › R-Code Simulation_Part1.zip!/Simulation_Part_1/Fig1_rev.pdf]

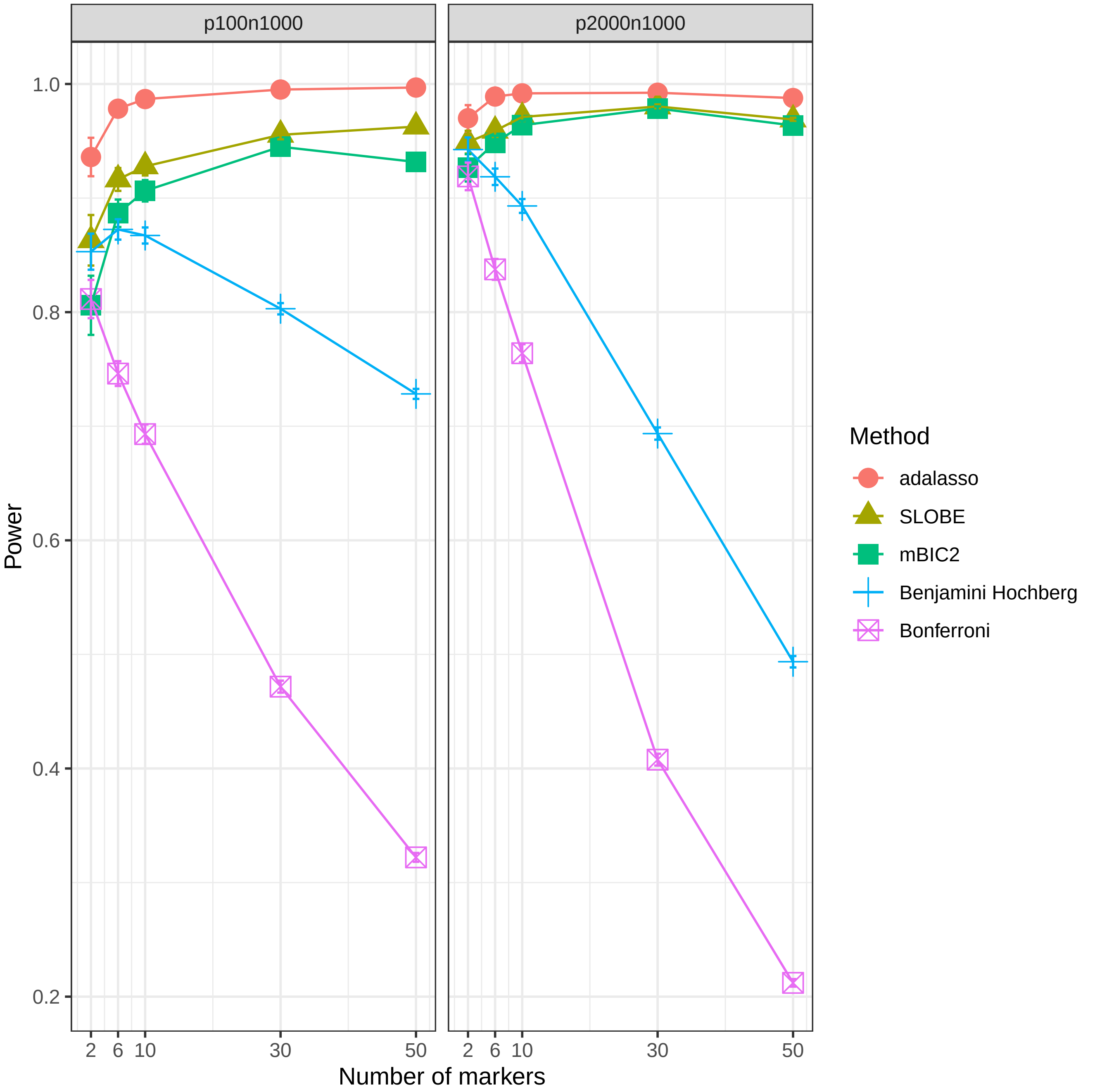

p100n1000
p2000n1000
1.0
0.8
0.6
0.4
0.2
Power
2
6
10
30
50
Number of markers
Method
adalasso
SLOBE
mBIC2
Benjamini Hochberg
Bonferroni
