# Supplementary material for: Selecting predictive biomarkers from genomic data
Source: PLoS One. 2022 Jun 16;17(6):e0269369. doi: 10.1371/journal.pone.0269369 (PMC9202896; doi:10.1371/journal.pone.0269369)
Supplement: S2 Data — The zip archive also includes R Markdown files which were used to obtain summary statistics from the simulation results and a README file which briefly indicates the organization of the simulation files. (ZIP) [file pone.0269369.s005.zip › R-Code Simulation_Part3.zip!/Simulation_SNP/Fig_indi_SNP.pdf]

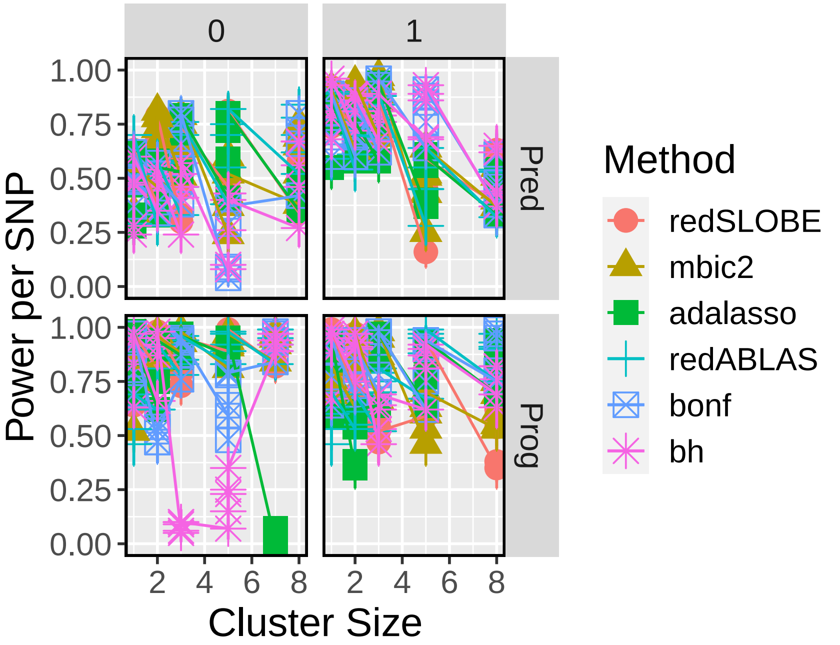
0
1
Pred
Prog
1.00
0.75
0.50
0.25
0.00
2
4
6
8
Power per SNP
Cluster Size
Method
redSLOBE
mbic2
adalasso
redABLAS
bonf
bh
